# PEPCOL: a GERCOR randomized phase II study of nanoliposomal irinotecan PEP02 (MM‐398) or irinotecan with leucovorin/5‐fluorouracil as second‐line therapy in metastatic colorectal cancer

**DOI:** 10.1002/cam4.635

**Published:** 2016-01-24

**Authors:** Benoist Chibaudel, Frédérique Maindrault‐Gœbel, Jean‐Baptiste Bachet, Christophe Louvet, Ahmed Khalil, Olivier Dupuis, Pascal Hammel, Marie‐Line Garcia, Mostefa Bennamoun, David Brusquant, Christophe Tournigand, Thierry André, Claire Arbaud, Annette K Larsen, Yi‐Wen Wang, C. Grace Yeh, Franck Bonnetain, Aimery de Gramont

**Affiliations:** ^1^ Department of Medical OncologyFranco‐British InstituteLevallois‐PerretFrance; ^2^ Groupe Coopérateur Multidisciplinaire en Oncologie (GERCOR)ParisFrance; ^3^ Preclinical and Translational Cancer Research UnitAAREC Filia Research (AFR)ParisFrance; ^4^ Department of Medical OncologySaint‐Antoine HospitalAssistance Publique des Hôpitaux de Paris (APHP)ParisFrance; ^5^ Department of GastroenterologyLa Pitié‐Salpetrière HospitalAssistance Publique des Hôpitaux de Paris (APHP)ParisFrance; ^6^ Department of Medical OncologyMontsouris Mutualiste InstituteParisFrance; ^7^ Department of Medical OncologyTenon HospitalAssistance Publique des Hôpitaux de Paris (APHP)ParisFrance; ^8^ Department of Medical OncologyVictor Hugo ClinicLe MansFrance; ^9^ Department of GastroenterologyBeaujon HospitalAssistance Publique des Hôpitaux de Paris (APHP)ClichyFrance; ^10^ Department of Medical OncologyHenri Mondor HospitalCréteilFrance; ^11^ Methodological and quality of life unit in oncology (EA3181) & Quality of life and cancer clinical research platformCHU BesançonBesançonFrance; ^12^ Cancer Biology and TherapeuticsINSERM U938 and Pierre and Marie Curie UniversityParisFrance; ^13^ PharmaEngine, Inc.TaipeiTaiwan

**Keywords:** Colorectal cancer, MM398, nanoliposomal irinotecan, PEP02, phase II

## Abstract

A multicenter, open‐label, noncomparative, randomized phase II study (PEPCOL) was conducted to evaluate the efficacy and safety of the irinotecan or PEP02 (MM‐398, nanoliposomal irinotecan) with leucovorin (LV)/5‐fluorouracil (5‐FU) combination as second‐line treatment in patients with metastatic colorectal cancer (mCRC). Patients with unresectable mCRC who had failed one prior oxaliplatin‐based first‐line therapy were randomized toirinotecan with LV/5‐FU (FOLFIRI) or PEP02 with LV/5‐FU (FUPEP; PEP02 80 mg/m^2^ with LV 400 mg/m^2^ on day 1 and 5‐FU 2400 mg/m^2^ on days 1–2). Bevacizumab (5 mg/kg, biweekly) was allowed in both arms. The primary endpoint was 2‐month response rate (RR). Fifty‐five patients were randomized (FOLFIRI,* n = *27; FUPEP,* n *= 28). In the intent‐to‐treat population (*n *= 55), 2‐month RR response rate was observed in two (7.4%) and three (10.7%) patients in the FOLFIRI and FUPEP arms, respectively. The most common grade 3–4 adverse events reported in the respective FOLFIRI and FUPEP arms were diarrhea (33% vs. 21%), neutropenia (30% vs. 11%), mucositis (11% vs. 11%), and grade 2 alopecia (26% vs. 25%). FUPEP has activity and acceptable safety profile in oxaliplatin‐pretreated mCRC patients.

## Introduction

The FOLFIRI regimen, combination of irinotecan with leucovorin (LV) and 5‐fluorouracil (5‐FU; LV/5‐FU)^1^ is a standard regimen in first‐line or second‐line therapy of metastatic colorectal cancer (mCRC) 2, 3.

PEP02 (MM‐398) is a highly stable nanoliposomal irinotecan that theoretically has therapeutic advantages over the free form of the drug (irinotecan and its active metabolite SN‐38) such as site‐specific delivery and extended release of drug. It was found to reduce the toxicity of the encapsulated agent to healthy tissue while maintaining or increasing its antitumor potency 4. Moreover, as compared to conventional irinotecan, PEP02 was associated with lower maximum concentration, longer elimination half‐life, higher area under the curve (AUC) for SN‐38, smaller volume of distribution, and slower plasma clearance of total irinotecan 4. In phase I studies, the maximum tolerated dose (MTD) of PEP02 as a single agent was 120 mg/m² once every 3 weeks and 80 mg/m² in combination with LV/5‐FU 5. A randomized phase II study of nanoliposomal irinotecan (PEP02) versus irinotecan versus docetaxel was conducted in advanced gastric cancer 6. The safety profile of PEP02 and irinotecan was similar, however, it was suggested that there may be an improvement of efficacy in a small subset of patients who received a slightly higher dose (150 mg/m^2^ every 3 weeks) of PEP02. The longer half‐life of PEP02 compared to irinotecan may potentiate the antitumor efficacy of 5‐FU.

This phase II study sought to evaluate the efficacy and safety of PEP02 in combination with LV/5‐FU, FUPEP regimen, as second‐line therapy in patients with mCRC.

## Material and Methods

### Design

PEPCOL (PEP for PEP02, the other denomination of MM‐398, COL for colorectal cancer) is a multicenter, noncomparative, open‐label, randomized phase II trial (EudraCT number: 2010–020468–39; ClinicalTrials.gov identifier: NCT01375816) in mCRC patients previously treated with an oxaliplatin‐based regimen. The study was conducted according to the International Conference on Harmonization Good Clinical Practice Guidelines, the Declaration of Helsinki, and the local regulatory requirements and laws. Written informed consents were obtained from all patients.

Patients were randomly assigned in a 1:1 ratio to either PEP02 plus LV/5‐FU (the FUPEP arm), or irinotecan plus LV5‐FU (the FOLFIRI arm), using a minimization technique with the three following stratification criteria: center, GERCOR prognostic score^7^ [Eastern Cooperative Oncology Group performance status (ECOG PS) 0, normal lactate dehydrogenase(LDH) value versus ECOG PS > 1, and/or LDH > 1 x Upper Normal Limit (ULN)], and first‐line time to progression (<9 months vs.* *≥ 9 months).

### Patient eligibility

Eligible patients were 18–75 years of age, had histologically confirmed adenocarcinoma of the colon or rectum, and documented measurable metastatic disease not suitable for curative surgery. Prior systemic oxaliplatin‐based first‐line therapy was required. Patients had to have an ECOG PS of 0–2 and adequate organ function (neutrophils 1.5 x 10^9^/L, platelets ≥100 x 10^9^/L, hemoglobin >9 g/dL, serum creatinine <150 *μ*mol/L, creatinine clearance >30 mL/min, and total bilirubin <1.5 x UNL). Exclusion criteria included preexisting (residual) diarrhea grade >1, total or partial bowel obstruction, prior chemotherapy with irinotecan, history or evidence of brain metastasis, exclusive bone metastasis upon physical examination, uncontrolled hypercalcemia, and pregnant or breast‐feeding women (Table S1).

### Treatment

The FUPEP regimen was administrated as follows: PEP02 80 mg/m^2^ intravenous (IV) over 90 min, with LV 400 mg/m^2^ IV over 2‐h followed by 5‐FU(5‐fluorouracil) 2400 mg/m^2^ continuous infusion over 46‐h. All treatment regimens were given every 14 days until occurrence of progressive disease (PD) or unacceptable toxicity. Two regimens of FOLFIRI were allowed: FOLFIRI‐1, irinotecan 180 mg/m^2^ IV over 90 min, with LV 400 mg/m^2^ IV over 2‐h, followed by 5‐FU 400 mg/m^2^ bolus and 5‐FU 2400 mg/m^2^ continuous infusion over 46‐h, and modified (m) FOLFIRI‐3, irinotecan 90 mg/m^2^ as 1‐h infusion, with LV 400 mg/m^2^ over 2‐h, followed by 5‐FU 2400 mg/m^2^ continuous infusion over 46‐h on day 1 and irinotecan 90 mg/m^2^ as 1‐h infusion repeated at the end of 5‐FU infusion on day 3. From June 2012, bevacizumab 5 mg/kg was added to the chemotherapy regimen.(Table S2) Premedication with atropine and antiemetics was permitted. Granulocyte colony‐stimulating factor was used according to the American Society of Clinical Oncology guidelines 8. Dose adjustments for each study treatment component individually and/or cycle delays were permitted in the event of toxicity. No crossover to FUPEP was permitted after progression in the FOLFIRI arm.

### End points

The primary endpoint was response rate (RR) evaluated at 2 months from randomization (2‐month RR) using RECIST version 1.1 9. Secondary endpoints were best objective RR (ORR) defined as the best response recorded from the start of the treatment until treatment failure, disease control rate (DCR) defined as the percentage of patients who have achieved a response or stabilization, overall survival (OS) defined as the time from the date of randomization to the date of patient death (from any cause) or to the last date the patient was known to be alive, progression‐free survival (PFS) defined as the time from the date of randomization to the date of progression (local, regional, or distant lesions) or death (from any cause). Alive patients without documented objective PD at the time of the final analysis were censored at the date of their last objective tumor assessment. Toxicity was evaluated according to the NCI‐CTCAE version 4.0.

Health‐related quality of life (HRQoL) assessments were performed in both arms at baseline, and after 4 and 8 cycles of treatment, using the French version of the EuroQol (EQ‐5D) and the Quality of Life QuestionnaireCore 30 (QLQ‐C30) 10. The EQ visual analog scale (VAS) of pain measure was also performed.

### Sample size

According to a Simon's Minimax two‐stage design 11 with a one‐sided 10% type I error, a power of 90% and a 15% improvement in 2‐month RR from 10% (H0, considered as uninteresting to pursue any further investigation) to 25% (H1, considered as promising to warrant further investigation in a phase III trial), 27 patients were required for the first stage and more than two responses per arm to proceed to the second stage of 44 patients in each arm, including a 10% drop‐out rate.

### Statistics

The primary analysis of efficacy used intent‐to‐treat (ITT) population, that is, including all randomized patients regardless of their eligibility and treatment received. The confirmative analysis was conducted in the modified ITT population of eligible patients and in a per‐protocol (PP) population comprising all patients who have received at least 2 cycles of the allocated treatment and without any major protocol deviations. The safety analysis included all patients who received at least one dose of any study drug. Follow‐up and survival were estimated using the reverse Kaplan–Meier method 12 and Kaplan–Meier method 13, respectively, and median values were described with 95% confidence intervals (CI).

The main clinical and medical patient characteristics were described based on the completion of at least one baseline HRQoL questionnaire. HRQoL baseline scores were described by treatment arm. Qualitative and continuous variables were described using percent and means (standard deviation) and medians (minimum‐maximum), respectively. The Mann–Whitney nonparametric test was used to compare HRQoL scores at baseline according to treatment arm. For exploratory purpose, a linear mixed‐effects (repeated measures of variance) model was used to analyze the longitudinal changes of HRQoL at baseline, and after 4 and 8 cycles of treatment. All patients who completed at least one baseline HRQoL assessment were included. Time, treatment, and interaction between time and treatment/performance status effects were explored in multivariate model. An unstructured covariance matrix for the individual random effects (individual deviance from average intercept) and time (individual deviance from average time effect) was employed.

## Results

### Patient population and treatment characteristics

**Figure 1:**
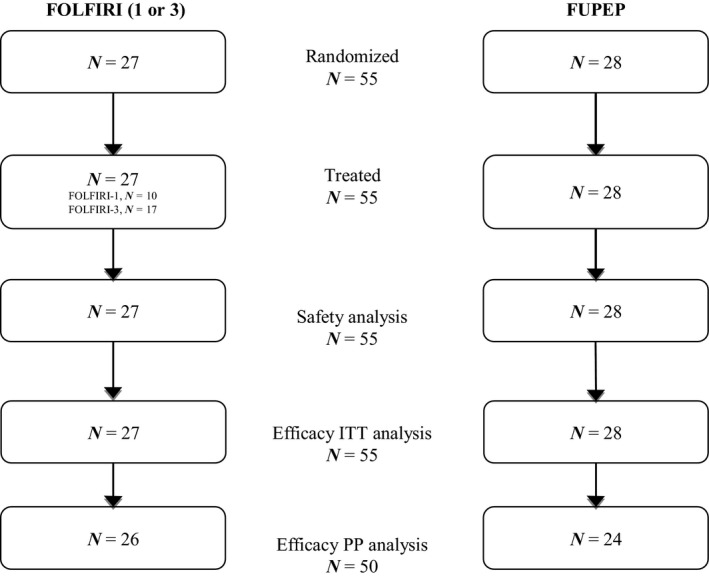
Participant flow.

Fifty‐five patients were randomized in six French centers from May 2011 to August 2013. Twenty‐seven patients were allocated to the FOLFIRI arm and 28 to the FUPEP arm (Fig. 1). The main patient and tumor characteristics are summarized in Table 1. Mean age was 62 years (range 35–77) in the FOLFIRI arm and 62 years (range 38–80) in the FUPEP arm. Population was balanced between the two arms.

**Table 1:** Baseline patient and disease characteristics

Variable	FOLFIRI (*N = *27)		FUPEP (*N = *28)	
	***N***	%	***N***	%
Age, years				
<70	22	81.5	22	78.6
≥70	5	18.5	6	21.4
Gender				
Male	14	51.8	19	67.9
Female	13	48.1	9	32.1
KRAS status				
Wild‐type	14	51.8	16	57.1
Mutated	10	37.0	10	35.7
Unknown	3	11.1	2	7.1
ECOG performance status				
0	15	55.6	10	35.7
1		40.7	14	50.0
2	111	3.7	4	14.3
BMI				
<25	17	63.0	17	60.7
25–30	9	33.3	6	21.4
≥30	1	3.7	5	17.9
Primary tumor location				
Colon	22	81.5	17	60.7
Rectum	5	18.5	10	35.7
Both	0	0	1	3.6
Primary tumor status				
Resected	21	77.8	19	67.9
Not resected	6	22.2	9	32.1
**Metastasis**				
Liver				
No	3	11.1	5	17.9
Yes	24	88.9	23	82.1
Lung				
No	12	44.4	17	60.7
Yes	15	55.6	11	39.3
Peritoneal				
No	17	63.0	23	82.1
Yes	10	37.0	5	17.9
Node				
No	23	85.2	20	71.4
Yes	4	14.8	8	28.6
Other tumor sites				
No	20	74.1	26	92.9
Yes	7	25.9	2	7.1
Number of metastatic sites				
1	12	44.4	12	42.9
2	4	14.8	14	50.0
3	10	37.0	0	0
4	1	3.7	2	7.1
Time to metastasis				
Synchronous	19	70.4	19	67.9
Metachronous	8	29.6	9	32.1
Prior adjuvant therapy				
No	22	81.5	22	78.6
Yes	5	18.5	6	21.4
First‐line PFS (months)				
>9	14	51.8	14	50.0
<9	13	48.1	14	50.0
Oxaliplatin failure after				
Adjuvant treatment	1	3.7	1	3.6
First‐line metastatic treatment	26	96.3	27	96.4
Oxaliplatin reintroduction before second‐line				
No	13	50.0	15	53.6
Yes	13	50.0	12	42.9
Missing data	1	3.7	1	3.6
Prior bevacizumab				
No	6	23.1	3	11.1
Yes	20	76.9	24	88.9
Missing data	1	3.7	1	3.6
White blood cell count				
<10000/mm^3^	27	100.00	27	96.4
≥10000/mm^3^	0	0	1	3.6
Neutrophils				
<4000/mm^3^	15	55.6	11	39.3
≥4000/mm^3^	12	44.4	17	60.7
Platelet				
<400000/mm^3^	25	92.6	24	85.7
≥400000/mm^3^	2	7.4	4	14.3
ALP				
Normal	8	29.6	13	46.4
1–3 × ULN	16	59.3	8	28.6
>3 × ULN	3	11.1	7	25.0
AST				
Normal	17	63.0	17	60.7
>1 × ULN	10	37.0	11	39.3
ALT				
Normal	20	74.1	23	82.1
>1 × ULN	7	25.9	5	17.9
Creatinine clearance				
≥60 mL/min	25	92.6	21	80.8
<60 mL/min	2	7.4	5	19.2
Missing data	0	0	2	7.1
CEA				
Normal	8	29.6	7	25.0
>1 × ULN	19	70.4	21	75.0
LDH				
Normal	10	37.0	9	34.6
>1 × ULN	16	59.3	17	65.4
Missing data	1	3.7	2	7.1
GERCOR prognostic model (20)				
Low‐risk	15	55.6	10	35.7
Intermediate risk	2	7.4	4	14.3
High‐risk	10	37.0	14	50.0
Chemotherapy regimen				
FOLFIRI‐1	10	37.0	0	0
mFOLFIRI‐3	17	63.0	0	0
FUPEP	0	0	28	100.0
Bevacizumab				
No	14	51.8	16	57.1
Yes	13	48.1	12	42.9

KRAS, Kirsten Rat Sarcoma viral oncogene homolog; BMI, Body mass index; ECOG, Eastern Cooperative Oncology Group; PFS, progression‐free survival; ALP, alkaline phosphatase; AST, aspartate aminotransferase; ALT, alanine transaminase; CEA, carcinoembryonic antigen; LDH, lactate dehydrogenase; ULN, upper limit normal.

In the FOLFIRI arm, 10 (37%) patients received FOLFIRI‐1 and 17 (63%) patients received mFOLFIRI‐3. All patients received at least one dose of the allocated study treatment. Bevacizumab was added to chemotherapy in 13 (48.1%) FOLFIRI‐treated patients and in 12 (42.9%) FUPEP‐treated patients.

The total number of cycles was 268 (range 1–22 cycles) in the FOLFIRI arm and 226 (range 1–25 cycles) in the FUPEP arm. The treatment was postponed by 35 (13.1%) and 18 (8.0%) cycles, in the FOLFIRI arm and FUPEP arm, respectively. The treatment dose was reduced in 33 (12.3%) cycles in the FOLFIRI arm and in 21 (9.3%) cycles in the FUPEP arm.

### Response rates

#### Tumor response rate at 2 months

At the end of the first step of the Simon's design, 2‐month RR was evaluated in the first 27 randomized patients in each arm (*n *= 54). A tumor response was observed in two (7.4%;95% CI: −2.5–17.3) and three (11.1%;95% CI: −0.7–22.2) patients in the FOLFIRI and FUPEP arms, respectively. In the ITT population, the 2‐month RR was 7.4% (*n* = 2/27) and 10.7% (*n* = 3/28).

#### Best overall response rate

**Figure 2:**
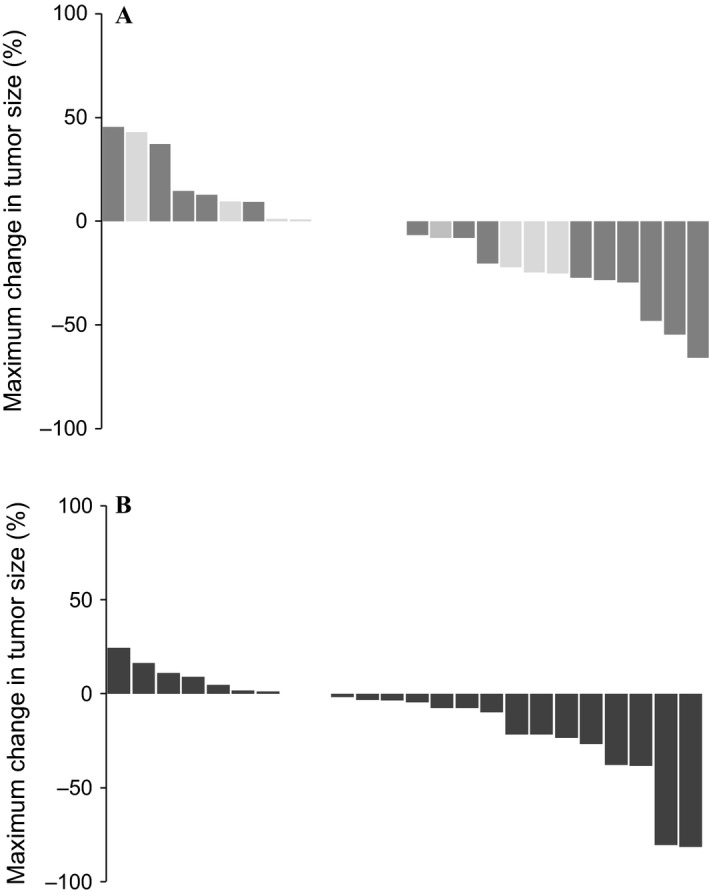
Waterfall plots showing the best response to the FOLFIRI arm (A) and the FUPEP arm (B) [arm A, light: FOLFIRI‐1; dark, mFOLFIRI‐3].

In the ITT population (*n *= 55), three (11.1%, 95% CI: 0.7–22.9) and four (14.3%, 95% CI: 1.3–27.3) patients had PR as the best response in the FOLFIRI and the FUPEP arm, respectively (Table 2). Tumor stabilization was observed in 17 (63.0%;95% CI: 44.8–81.2) patients in the FOLFIRI arm and 13 (46.4%;95% CI: 27.9–64.9) patients in the FUPEP arm. PD at first evaluation was demonstrated in six (22.2%;95% CI: 6.5–37.9) patients in the FOLFIRI arm and seven (25.0%;95% CI: 9.0–41.0) patients in the FUPEP arm. No CR was observed. (Fig. 2) Of note, all responses in the FOLFIRI arm were reported in patients having received the mFOLFIRI‐3 regimen (ORR = 0.0% for FOLFIRI‐1 vs. ORR = 17.6% for mFOLFIRI‐3). The DCR was 74.1% (20/27) in the FOLFIRI arm and 60.7% (17/28) in the FUPEP arm.

Five patients were excluded from PP analysis due to early death (lung infection without neutropenia one patient, cancer one patient), limiting diarrhea (two patients), and PEP02‐related allergy (one patient). Of the 50 evaluable patients (26 FOLFIRI‐treated patients, 24 FUPEP‐treated patients), PR was observed in three (11.5%; 95% CI: 0.7–23.8) patients in the FOLFIRI arm and four (16.7%; 95% CI: 1.8–31.6) patients in the FUPEP arm Table 2.

**Table 2:** Best overall response rate (ITT population, *N* = 55; PP population, *N* = 50)

	FOLFIRI		FUPEP	
	*N* (%)	95% CI	*N* (%)	95% CI
Best ORR				
ITT	3/27 (11.1)	−0.74–22.97	4/28 (14.3)	1.32–27.25
PP	3/26 (11.5)	−0.74–23.82	4/24 (16.7)	1.76–31.58
2‐month ORR				
ITT	2/27 (7.4)	−2.47–17.29	3/28 (10.7)	−0.74–22.17
PP	2/26 (7.7)	−2.55–17.94	3/24 (12.5)	−0.73–25.73

ORR: overall response rate, ITT, intent to treat; PP, per‐protocol.

### Survivals

After a median follow‐up of 11.6 months (95% CI: 10.2–19.8), 35 patients progressed and 33 patients died. The median PFS was 6.8 months (95% CI: 3.7–8.1) in the FOLFIRI arm and 5.0 months(95% CI: 2.8–6.0) in the FUPEP arm. The median OS was 10.5 months (95% CI: 6.9–21.1) in the FOLFIRI arm and 14.6 months(95% CI: 6.9–16.5) in the FUPEP arm.

### Safety

The most frequent grade 3–4 toxicity in the respective FOLFIRI and FUPEP arms were diarrhea (33.3% vs. 21.4%), neutropenia (29.6% vs. 10.7%), grade 2 alopecia (25.9% vs. 25.0%), stomatitis (11.1% vs. 10.7%), and nausea (7.4% vs. 3.6%) (Table 3). There was no toxic‐related death in either of the arms. The addition of bevacizumab to both regimens did not significantly increase grade 3–4 toxicities (Table S3).

Twenty‐three serious adverse events (SAE) were reported during the study (13 in the FOLFIRI arm and 10 in the FUPEP arm). In the FUPEP arm, six SAEs were related to PEP02 (two severe diarrhea, two allergic reactions, one ileitis, and one general state alteration).

### Subsequent therapy

Two (7.4%) patients had salvage surgery for metastasis with complete tumor resection (R0) in the FOLFIRI arm. None of the FUPEP‐treated patients underwent surgery.

After study treatment, 44 patients received third‐line therapy (19 in the FOLFIRI arm and 25 in the FUPEP arm). Irinotecan‐based therapy as third‐line treatment after FUPEP was administered in 21 patients, either in combination with fluoropyrimidine (*n *= 12) or an anti‐EGFR agent (*n *= 9). Two PRs were observed with the combination of panitumumab and irinotecan.

### Health‐related quality of life

There was no significant difference at baseline between two treatment arms regarding all the dimension of both questionnaires. The mean EQ‐5D global health status (GHS)/VAS scores in the FOLFIRI arm were stable between baseline and eight cycles of treatment (scores: 67.7–67.5), but higher compared with the FUPEP arm (scores: 61.5–58.0). The QLQ‐C30 GHS/HRQoL scores at baseline were similar between the two treatment arms (65.4 in the FOLFIRI arm and 65.7 in the FUPEP arm).

The FUPEP arm presented a better HRQoL level at baseline compared with the FOLFIRI arm that was characterized by higher scores for three functional scores (emotional, social, and physical functioning), and less pain. On contrary, FUPEP‐treated patients had a higher fatigue score compared to those treated with FOLFIRI.

Longitudinal analysis of the EQ‐5D and QLQ‐C30 scores were performed for 45 and 48 patients, respectively. No significant treatment arm effects on any functional or symptom scores were observed. In the multivariate model, the time effect of emotional functioning, diarrhea, and the time‐treatment interaction on physical functioning were significant. FUPEP‐treated patients had more diarrhea and less emotional functioning abilities.

No differences were observed for GHS over time between the two arms, but patients in the FUPEP arm presented a higher deterioration of the physical functioning and more fatigue.

## Discussion

This is the first randomized phase II study evaluating the effect of adding PEP02 to LV/5‐FU when administered in mCRC patients who failed prior oxaliplatin‐based first‐line therapy. In the ITT population, 2‐month RR was similar in both arms (7.4% vs. 10.7%). According to the Simon's Minimax decision rules, the targeted RR was reached only in the FUPEP arm, but not in the FOLFIRI arm. Despite a potential higher antitumor activity than that of the widely used FOLFIRI‐1 regimen, it is unlikely that FUPEP could challenge the mFOLFIRI‐3 efficacy with the data reported here. This is the main reason why GERCOR (sponsor of the study) decided not to proceed to the second stage of the study, but to make an attempt to optimize the FUPEP regimen. Of note, RR of the FUPEP regimen (14.3%) was closer to that of mFOLFIRI‐3 (17.6%) than to FOLFIRI‐1 (0%). (Fig. 2) In previous studies which evaluated the FOLFIRI‐3 regimen as second‐line treatment in mCRC patients, RR has ranged between 7.4% and 23.0% without bevacizumab and 22.4% and 35.0% when adding bevacizumab (Table S4).

The FUPEP combination safety profile remains similar to that of FOLFIRI, with diarrhea being the most significant SAE (21% in the FUPEP arm, 30% in the FOLFIRI arm) and the incidence of severe neutropenia being around 11% (compared to 30% with free irinotecan). Yet, no unexpected toxicities were observed. Of note, the addition of bevacizumab did not lead to the increased incidence of adverse events.

Based on the preliminary results of the PEPCOL study, the FUPEP regimen was added as the third arm to the positive phase III trial of metastatic pancreatic cancer patients previously treated with gemcitabine‐based therapy (NAnoliPOsomaL Irinotecan, NAPOLI‐(1). FUPEP was found superior to 5FU 14, 15.

In colorectal cancer, the results of the PEPCOL study suggest that the FUPEP regimen could be as active as the optimized mFOLFIRI3 regimen, but more active than the standard FOLFIRI regimen in oxaliplatin‐pretreated mCRC patients with an acceptable safety profile. FUPEP may also safely be combined with bevacizumab. With further ongoing optimization, this regimen has the potential to provide a clinically useful treatment for post‐oxaliplatin mCRC patients Table 3.

**Table 3:** Incidence of adverse events observed per patient (***N*** = 55)

	FOLFIRI (*N* = 27)										FUPEP (*N* = 28)									
NCI‐CTCAE grade	0		1		2		3		4		0		1		2		3		4	
	***N***	%	***N***	%	***N***	%	***N***	%	***N***	%	***N***	%	***N***	%	***N***	%	***N***	%	***N***	%
Neutropenia	9	33.3	4	14.8	6	22.2	6	22.2	2	7.4	15	53.6	6	21.4	4	14.3	2	7.1	1	3.6
Anemia	3	11.1	18	66.7	5	18.5	1	3.7	0	0.0	6	21.4	18	64.3	4	14.3	0	0.0	0	0.0
Thrombocytopenia	18	66.7	8	29.6	1	3.7	0	0.0	0	0.0	20	71.4	8	28.6	0	0.0	0	0.0	0	0.0
Diarrhea	3	11.1	7	25.9	8	29.6	9	33.3	0	0.0	7	25.0	5	17.9	10	35.7	4	14.3	2	7.1
Nausea	7	25.9	13	48.1	5	18.5	2	7.4	0	0.0	6	21.4	10	35.7	11	39.3	1	3.6	0	0.0
Vomiting	18	66.7	5	18.5	3	11.1	1	3.7	0	0.0	13	46.4	4	14.3	10	35.7	1	3.6	0	0.0
Mucositis/stomatitis	12	44.4	8	29.6	4	14.8	3	11.1	0	0.0	13	46.4	11	39.3	1	3.6	3	10.7	0	0.0
Alopecia	7	25.9	13	48.1	7	25.9	–		–		14	50.0	7	25.0	7	25.0	–		–	

NCI‐CTCAE, the National Cancer Institute‐Common Toxicity Criteria Adverse Events.

## Conflict of Interest

BC has acts as consultant to Sanofi, and has received honoraria from Roche and Sanofi. AdG acts as a consultant to PharmaEngine, Inc, and serves as a consultant on advisory boards to Roche. JBB acts as a consultant to Amgen, Celgene, Merck Serono, and Sanofi, and has received honoraria from Bayer, Lilly, and Roche. AKL has received research funding from Merrimack. FB acts as a consultant to Novartis, Roche, EISAI, Integragen, Invecty, and Nestlé, and has received honoraria from Roche, Celgene, and Merck Serono, and a grant from Roche. CT served as a consultant on advisory board to PharmaEngine Inc. TA acts as a consultant to Roche and has received honoraria from Roche. YWW is employee of PharmaEngine Inc, and has an ownership interest in PhamraEngine Inc. CGY is employee of PharmaEngine Inc., has been compensated for a leadership role by PharmaEngine Inc, and has an ownership interest in PhamraEngine Inc. All remaining authors have declared no conflicts of interest.

## Supporting information


**Table S1.** List of all eligibility criteria.

**Table S2.** Outline of the study schedule and treatment regimens: FOLFIRI‐1 (A), mFOLFIRI‐3 (B), and FUPEP (C).

**Table S3.** NCI grade 3/4 toxicity in the FOLFIRI arm and in the FUPEP arm according to bevacizumab use

**Table S4.** Trials assessing the efficacy and safety profiles of the FOLFIRI (1 or 3) regimen with and without bevacizumab as second‐line therapy in patients with metastatic colorectal cancer.
