# Supplementary material for: PEPCOL: a GERCOR randomized phase II study of nanoliposomal irinotecan PEP02 (MM‐398) or irinotecan with leucovorin/5‐fluorouracil as second‐line therapy in metastatic colorectal cancer
Source: Cancer Med. 2016 Jan 24;5(4):676–83. doi: 10.1002/cam4.635 (PMC4831286; doi:10.1002/cam4.635)
Supplement: Supplementary file 1 — Table S1. List of all eligibility criteria. [file CAM4-5-676-s001.docx]

**Table S1.** List of all eligibility criteria

| *Inclusion criteria* |
| --- |
| Patients had to fulfill all of the following criteria to be eligible:   - Signed informed consent before proceeding with any study procedure, - Histologically proven adenocarcinoma of the colon or rectum, - Metastatic disease confirmed according to RECIST version 1.1, - Duly documented inoperable metastatic disease, i.e. not suitable for complete carcinological surgical resection, - Previous oxaliplatin-based chemotherapy for metastatic disease, - Patients regardless of KRAS status (wild-type or mutated) or previous anti-EGFR treatment, - Measurable lesion (≥1 cm) as assessed by CT-scan or magMRI according to RECIST criteria (version 1.1), - Age 18 -75 years, - WHO ECOG performance status (ECOG PS) 0-2, - Hematological status: neutrophils ≥1.5x10^9^/L; platelets ≥100 x10^9^/L, and hemoglobin >9 g/dL (may be transfused to maintain or exceed this level), - International Normalized Ratio (INR) ≤1.5 [patients on full anticoagulation due to venous thromboembolism must have their INR in-range of 2 and 3; any anticoagulation therapy must be at stable dosing prior to treatment start]; activated partial thromboplastin time <1.5x Upper Normal Limit (UNL), - Adequate renal function: serum creatinine level <150 μmol/L and calculated creatinine clearance level >30 ml/min[calculated according to the Modification of Diet in Renal Disease (MDRD) formula], - Adequate hepatic function: total bilirubin <1.5 x UNL, - Proteinuria <2+ (dipstick urinalysis) or ≤1g/24h, - Regular follow‑up feasible; a registered patient must be treated and followed at the participating center, - Baseline evaluations performed before randomization: clinical and blood evaluations no more than 2 weeks prior to randomization; tumoral assessment (chest X-ray, CT scan, or MRI, evaluation of non-measurable lesions) no more than 21 days prior to randomization, - First course of treatment planned less than 7 days after randomization,   - In pre-menopausal women and women <2 years after the onset of menopause, a negative serum pregnancy test within 7 days before starting study treatment;  - Fertile women and men of childbearing potential (<2 years after last menstruation in women) must use effective means of contraception (oral contraceptives, intrauterine contraceptive device, barrier method of contraception in conjunction with spermicidal jelly, or surgical sterilization),   - Registration in France with the French National Health Care System [including “couverture maladie universelle” (CMU)]. |
| *Exclusion criteria* |
| - Severe arterial thromboembolic events (myocardial infarction, stroke) less than 6 months before inclusion, - Baseline diarrhea grade >1, - Total or partial bowel obstruction, - Pregnant or breast-feeding women, - Previous chemotherapy with irinotecan, - History or evidence of central nervous system metastasis upon physical examination, - Exclusive bone metastasis, - Uncontrolled hypercalcemia, - Uncontrolled hypertension (defined as persistent systolic blood pressure >150 mmHg and/or diastolic blood pressure >100 mmHg), or medical history of hypertensive crisis, or hypertensive encephalopathy, - Other concomitant or previous malignancy, except adequately treated in situ carcinoma of the uterine cervix, basal or squamous cell carcinoma of the skin, or cancer in complete remission for < 5 years, - Major surgery or traumatic injury within the last 28 days prior to randomization, - Concomitant antitumoral treatment, other than those planned in the study protocol, - Participation in another clinical trial with any investigational drug within 30 days prior to randomization, - Symptomatic ascities or pleural effusion not evacuated prior to entry into the study, - Other serious and uncontrolled non-malignant disease, - Patients with known allergy to any excipients of the study drugs. |
