# Supplementary material for: PEPCOL: a GERCOR randomized phase II study of nanoliposomal irinotecan PEP02 (MM‐398) or irinotecan with leucovorin/5‐fluorouracil as second‐line therapy in metastatic colorectal cancer
Source: Cancer Med. 2016 Jan 24;5(4):676–83. doi: 10.1002/cam4.635 (PMC4831286; doi:10.1002/cam4.635)
Supplement: Supplementary file 2 — Table S2. Outline of the study schedule and treatment regimens: FOLFIRI‐1 (A), mFOLFIRI‐3 (B), and FUPEP (C). [file CAM4-5-676-s002.doc]

**Table S2.** Outline of the study schedule and treatment regimens. (A) FOLFIRI-1 regimen, (B) mFOLFIRI-3 regimen, and (C) FUPEP regimen

FOLFIRI-1

mFOLFIRI-3

FUPEP

**Folinic Acid**

400mg/m²

**5-FU continuous infusion**

2400mg/m² /46h

**CPT11**

180mg/m²

**Folinic Acid**

400mg/m²

**5-FU continuous infusion**

2400mg/m² /46h

**CPT11**

90mg/m²

**CPT11**

90mg/m²

H_0_

H_+2_

H_+24_

H_+48_

**Folinic Acid**

400mg/m²

**5-FU continuous infusion**

2400mg/m² /46h

**PEP02**

80mg/m²

**A**

**B**

**C**

Blue arrow, 5-FU bolus (400mg/m² / 15min)
