# Supplementary material for: PEPCOL: a GERCOR randomized phase II study of nanoliposomal irinotecan PEP02 (MM‐398) or irinotecan with leucovorin/5‐fluorouracil as second‐line therapy in metastatic colorectal cancer
Source: Cancer Med. 2016 Jan 24;5(4):676–83. doi: 10.1002/cam4.635 (PMC4831286; doi:10.1002/cam4.635)
Supplement: Supplementary file 3 — Table S3. NCI grade 3/4 toxicity in the FOLFIRI arm and in the FUPEP arm according to bevacizumab use [file CAM4-5-676-s003.docx]

**Table S3.** NCI-CTCAE grade 3/4 toxicity in the FOLFIRI arm and in the FUPEP arm according to the use of bevacizumab

|  | **FOLFIRI**  **(*N* = 14)** | | **FOLFIRI/**  **bevacizumab**  **(*N* = 13)** | | **FUPEP (*N* = 16)** | | **FUPEP/**  **bevacizumab**  **(*N* = 12)** | |
| --- | --- | --- | --- | --- | --- | --- | --- | --- |
|  | *N* | % | *N* | % | *N* | % | *N* | % |
| **Hematologic** |  |  |  |  |  |  |  |  |
| Neutropenia | 4 | 28.6 | 4 | 30.8 | 1 | 6.2 | 2 | 16.7 |
| Anemia | 0 | 0 | 1 | 7.7 | 0 | 0 | 0 | 0 |
| Thrombocytopenia | 0 | 0 | 0 | 0 | 0 | 0 | 0 | 0 |
| **Non-hematologic** |  |  |  |  |  |  |  |  |
| Diarrhea | 3 | 21.4 | 6 | 46.1 | 2 | 12.5 | 4 | 33.3 |
| Nausea | 2 | 14.3 | 0 | 0 | 0 | 0 | 1 | 8.3 |
| Vomiting | 1 | 7.1 | 0 | 0 | 1 | 6.2 | 0 | 0 |
| Stomatitis | 1 | 7.1 | 2 | 15.4 | 1 | 6.2 | 2 | 16.7 |
| Alopecia (grade 2) | 4 | 28.6 | 3 | 23.1 | 5 | 31.2 | 2 | 16.7 |
