# Supplementary material for: PEPCOL: a GERCOR randomized phase II study of nanoliposomal irinotecan PEP02 (MM‐398) or irinotecan with leucovorin/5‐fluorouracil as second‐line therapy in metastatic colorectal cancer
Source: Cancer Med. 2016 Jan 24;5(4):676–83. doi: 10.1002/cam4.635 (PMC4831286; doi:10.1002/cam4.635)
Supplement: Supplementary file 4 — Table S4. Trials assessing the efficacy and safety profiles of the FOLFIRI (1 or 3) regimen with and without bevacizumab as second‐line therapy in patients with metastatic colorectal cancer. [file CAM4-5-676-s004.doc]

**Supplementary Table S4** Trials assessing the efficacy and safety profiles of the FOLFIRI (1 or 3) regimen with and without bevacizumab as second-line therapy in patients with metastatic colorectal cancer

|  |  |  | **Efficacy** | | |  | **Grade 3-4 toxicity** | | |
| --- | --- | --- | --- | --- | --- | --- | --- | --- | --- |
| **First author, study** | **Regimen** | ***N*** | **RR**  **%** | **PFS**  **months** | **OS**  **months** |  | **Neutropenia**  **%** | **Diarrhea**  **%** | **Mucositis**  **%** |
| Tournigand *et al*, 2004 | FOLFIRI-1 | 69 | 4.0 | 2.5 | - |  | 21 | 8.0 | 3.0 |
| Muro *et al*, 2010 | FOLFIRI-1 | 213 | 16.7 | 5.1 | 18.2 |  | 52.1 | 4.7 | 0.5 |
| Hirata *et al*, 2014 | FOLFIRI-1 | 50 | 12.0 | 5.8 | - |  | 30.0 | 0.5 | 1.5 |
| Peeters *et al*, 2010 |  |  |  |  |  |  |  |  |  |
| Wild-type KRAS | FOLFIRI-1 | 294 | 10.0 | 3.9 | 12.5 |  | 23 | 9.0 | 3.0 |
| Mutated KRAS | FOLFIRI-1 | 248 | 14.0 | 4.9 | 11.1 |  | 17 | 11.0 | 4.0 |
| Van Cutsem *et al*, 2012 | FOLFIRI-1 | 612 | 11.1 | 4.7 | 12.1 |  | 29.5 | 7.8 | 5.0 |
| Mabro *et al*, 2006 | FOLFIRI-3 | 65 | 23.0 | 4.7 | 10.5 |  | 11.0 | 23.0 | 7.0 |
| Bidard *et al*, 2009 | FOLFIRI-3 | 109 | 17 | 3.7 | 9.3 |  | - | - | - |
| Viel *et al*, 2008 | FOLFIRI-3 | 27 | 7.4 | 4.5 | 8.9 |  | 7.0 | 11.0 | - |
| Beretta *et al*, 2013 | FOLFIRI-1/bevacizumab | 435 | 26 | 8.3 | 17.2 |  | - | - | - |
| Ghiringhelli *et al*, 2012 | FOLFIRI-3/bevacizumab | 49 | 22.4 | 7.0 | 13.0 |  | 22.0 | 16.0 | 2.0 |
| Bourges *et al*, 2009 | FOLFIRI-3/bevacizumab | 31 | 35.0 | 6.2 | 10.8 |  | 7.4 | 32.1 | 10.7 |
| Chibaudel B, present study | FOLFIRI-1  mFOLFIRI-3  FUPEP | 10  17  28 | 0.0  17.6  14.3 | 5.7  7.6  5.0 | 10.2  12.2  14.6 |  | 40.0  23.6  10.7 | 30.0  35.3  21.4 | 10.0  11.8  10.7 |

KRAS: Kirsten Rat Sarcoma viral oncogene homolog; RR: response rate; PFS: progression-free survival; OS, overall survival
